# Transcriptomic Analysis of Gill and Kidney from Asian Seabass (*Lates calcarifer*) Acclimated to Different Salinities Reveals Pathways Involved with Euryhalinity

**DOI:** 10.3390/genes11070733

**Published:** 2020-06-30

**Authors:** Shubha Vij, Kathiresan Purushothaman, Prakki Sai Rama Sridatta, Dean R. Jerry

**Affiliations:** 1 Reproductive Genomics Group, Temasek Life Sciences Laboratory, Singapore 117604, Singapore; kathiresan.purushothaman@nord.no (K.P.); prakki_sr_sridatta@ncid.sg (P.S.R.S.); 2 School of Applied Science, Republic Polytechnic, 9 Woodlands Avenue 9, Singapore 738964, Singapore; 3 Faculty of Biosciences and Aquaculture, Nord University, 8049 Bodø, Norway; 4 Infectious Disease Research Laboratory, National Centre for Infectious Diseases (NCID), Tan Tock Seng Hospital, Singapore 308442, Singapore; 5 Tropical Futures Institute, James Cook University, Singapore 387380, Singapore; 6 Centre for Sustainable Tropical Fisheries and Aquaculture, James Cook University, Townsville 4810, Australia

**Keywords:** euryhalinity, Asian seabass, fish, osmoregulation, acclimation, transcriptome, pliable trait, freshwater transcriptome, marine transcriptome

## Abstract

Asian seabass (or commonly known as barramundi), *Lates calcarifer,* is a bony euryhaline teleost from the Family *Latidae*, inhabiting nearshore, estuarine, and marine connected freshwaters throughout the tropical Indo-West Pacific region. The species is catadromous, whereby adults spawn in salinities between 28 and 34 ppt at the mouth of estuaries, with resultant juveniles usually moving into brackish and freshwater systems to mature, before returning to the sea to spawn again as adults. The species lives in both marine and freshwater habitats and can move quickly between the two; thus, the species’ ability to tolerate changes in salinity makes it a good candidate for studying the salinity acclimation response in teleosts. In this study, the transcriptome of two major osmoregulatory organs (gills and kidneys) of young juvenile Asian seabass reared in freshwater and seawater were compared. The euryhaline nature of Asian seabass was found to be highly pliable and the moldability of the trait was further confirmed by histological analyses of gills and kidneys. Differences in major expression pathways were observed, with differentially expressed genes including those related to osmoregulation, tissue/organ morphogenesis, and cell volume regulation as central to the osmo-adaptive response. Additionally, genes coding for mucins were upregulated specifically under saline conditions, whereas several genes important for growth and development, as well as circadian entrainment were specifically enriched in fish reared in freshwater. Routing of the circadian rhythm mediated by salinity changes could be the initial step in salinity acclimation and possibly migration in euryhaline fish species such as the Asian seabass.

## 1. Introduction

Fishes are the most speciose amongst the vertebrates, represented by more than 33,000 species [1]. The water environment of fishes is usually defined in terms of salinity as either freshwater (<0.5 parts per thousand (ppt)), brackish 0.5–30 ppt, or marine (30–40 ppt) [2]. The large majority of teleosts are stenohaline and only able to survive in habitats with relatively stable salinity. Thus, such fishes can live either exclusively in freshwater (e.g., goldfish, guppies, and channel catfish), or in marine environments (e.g., pufferfish, tuna, swordfish) [2]. However, there are also a minority of fish species (2–3%) that are euryhaline (*eurys*: broad; *halinos*: salinity) and capable of tolerating broad fluctuations in salinities. Euryhaline fishes typically inhabit estuaries, where salinity changes are frequent, or are often migratory and capable of staying both in fresh and seawater during different stages of the life-cycle. The migration can be either towards the freshwater to breed (anadromous, e.g., *Salmo salar*, *Gasterosteus aculeatus*, *Acipenser medirostris*, *Petromyzon marinus*), or the ocean (catadromous, e.g., *Anguilla rostrata*, *Anguilla japonica*, *L. calcarifer*). The existence of diadromy (movement of fish between fresh and marine environment to complete their lifecycle) is phylogenetically primitive, indicating that this life-history represents an evolutionarily ancient strategy [2]. Further, in spite of the obvious evolutionary advantage of euryhalinity (in terms of fish survival in the face of salinity fluctuations), the infrequent occurrence of euryhaline fishes indicates the considerably high costs associated with the physiological plasticity [2,3] and the trait would have been lost, if not for strong selection pressure.

Euryhaline fish are adapted to deal with the osmoregulatory consequences of disparate saline environments, although the exact physiological and gene expression mechanisms allowing them to maintain homeostasis and tolerate salinity fluctuations are not completely understood. Osmolarity of fish blood plasma usually ranges between 300 and 400 mOsmL^−1^, while dependent on the salinity of the water in which they live, osmotic pressures in the external medium may be as low as 0 mOsmL^−1^ in fresh water (hyposmotic) to 1100 mOsmL^−1^ in seawater (hyperosmotic) [4]. In the hyperosmotic marine environment, fishes tend to gain salts and lose water by passive diffusion, while the opposite happens in freshwater with fishes gaining water and losing salts [5]. Therefore, in the hyposmotic freshwater environment, homeostasis is achieved by restricting the water influx through the body surface and by expelling water excessively through the kidneys [6]. The gills and skin in freshwater fishes actively uptake ions from the water to make up for the unavoidable loss of ions excreted with the water. For example, freshwater fishes have specialized epithelial cells termed ionocytes in the gill and skin which actively maintain osmotic homeostasis by absorbing salt from the external environment. Conversely, in the marine environment, acclimation of fish includes drinking large amounts of water, expelling excess salts, and increased water absorption through the gut. Therefore, central to the acclimation in euryhaline fishes is the ability to reverse the drinking rate, as well as ion fluxes, to survive in different salinities [2,7].

In order to be euryhaline, fish osmoregulatory organs such as gills and kidneys are required to alter the relevance of cell types together with the intracellular landscape to adapt to changing ionic and osmotic environments. This versatility enables the fish to move from freshwater to seawater and vice-versa [8]. In fishes, gills play an important role in maintaining osmolarity in the organism, serving to passage the ions and water molecules to-and-from the fish body and its environment. The gill epithelium is mainly comprised of non-differentiated, neuroepithelial, chloride, mucous, and pavement cells. The chloride cells, also called ionocytes, are specialized cells which are rich in mitochondria and have ion transporters and channels embedded in the plasma membrane. These cells play a key role in maintaining the iono-osmotic balance by essentially serving to counter the effect of the environment; absorbing (sequestering) ions in freshwater and flushing (secreting) ions in the ‘high salt’ marine environment. Another important osmoregulatory organ is the kidney which also plays an important role in keeping the water–salt balance in fishes [9].

Small-scale studies have reported various aspects of fish euryhalinity, including expression analyses of a set of targeted key genes, such as genes involved in iono-osmoregulation (European eel *Anguilla anguilla*, Japanese eel *Anguilla japonica*, and killifish *Fundulus heteroclitus*) [10,11,12,13,14]. Understandably, as only a small subset of possible genes involved in iono-osmoregulation were targeted, these have provided limited insights into the response of the transcriptome of euryhaline fish when exposed to different salinities. However, recently, studies have focused on obtaining a global view of the euryhaline fish transcriptome. These include microarray-based transcriptome profiling in the euryhaline longjaw mudsucker (*Gillichthys mirabilis*) and killifish (*Fundulus majalis* and *F. heteroclitus*), as well as Next Generation Sequencing (NGS)-based studies in amphidromous ayu (*Plecoglossus altivelis*), Mozambique tilapia (*Oreochromis mossambicus*), threespine stickleback (*G. aculeatus*), Alewife (*Alosa pseudoharengus*), and Sacramento splittail (*Pogonichthys macrolepidotus*), [8,15,16,17,18,19,20]. Both small-scale and large-scale studies have shown that major responses to salinity occur at the transcriptome level, coupled with extensive cellular remodeling, and have identified differential expression of ion transporters as the key step instrumental in restoring homeostasis. In addition, compensatory acclimation includes changes in pathways related to hormones, energy metabolism, alteration of cell structure and function, as well as other genes important for restoring osmotic homeostasis [8,21,22,23,24,25,26,27,28,29].

Due to their ability to adapt to varying salinities, euryhaline fishes provide an excellent model to understand the genetic basis of salt acclimation in the aquatic environment [30,31,32,33]. Asian seabass (*L. calcarifer*; Bloch, 1790), also known as barramundi or giant perch, is an euryhaline, carnivorous teleost, popular as a food fish in southeast Asia, Australia, and most of the Indian subcontinent [34,35,36,37,38,39]. Asian seabass is catadromous (fish that spend most of their life in freshwater, but spawn in seawater), although Asian seabass do not necessarily always migrate [39]. The species as adults spawn in near marine conditions (28–34 ppt) at the mouth of estuaries and rocky headlands, with resultant larvae usually entering brackish estuaries to metamorphose into juveniles in seagrass beds or coastal swamps. Juveniles then spend the majority of their life in fresh and/or brackish waters, before migrating to the marine environment to breed [40]. Thus, under natural conditions, the life cycle of this species is complex including freshwater, estuarine, and marine phases [40,41,42,43]. Asian seabass can also rapidly acclimate to different salinities, including from freshwater to full marine and vice versa in a few hours, and in aquaculture often juveniles bred and held in saltwater are rapidly acclimated to freshwater for farming (or vice-versa depending on nursery stage and production system) [39,44].

The aim of this study was to decipher the molecular basis of salinity acclimation in this euryhaline species. Gills and kidneys, the two major osmoregulatory (as well as excretory) organs which help the fish to adapt to varying salinities were the focus of this study. Therefore, we obtained the global gill and kidney transcriptome of juvenile Asian seabass reared in either freshwater (0 ppt), marine conditions (32 ppt), or reared initially in freshwater and then transferred into seawater (as occurs regularly in aquaculture production; [39]). We also performed a histological analysis in order to ascertain the effect of changed salinity conditions in these two tissues of juvenile Asian seabass.

## 2. Materials and Methods

### 2.1. Ethics Statement

All experiments were approved by Agri-food and Veterinary Authority (AVA) Institutional Animal Care and Use Committee (IACUC) (approval ID: AVA-MAC-2012-02), Temasek Life Sciences Laboratory (TLL) Institutional Animal Care and Use Committee (IACUC) (approval ID: TLL (F)-14-003) and performed according to guidelines set by the National Advisory Committee on Laboratory Animal Research (NACLAR) for the care and use of animals for scientific research in Singapore.

### 2.2. Experimental Setup and Sampling

**Figure 1:**
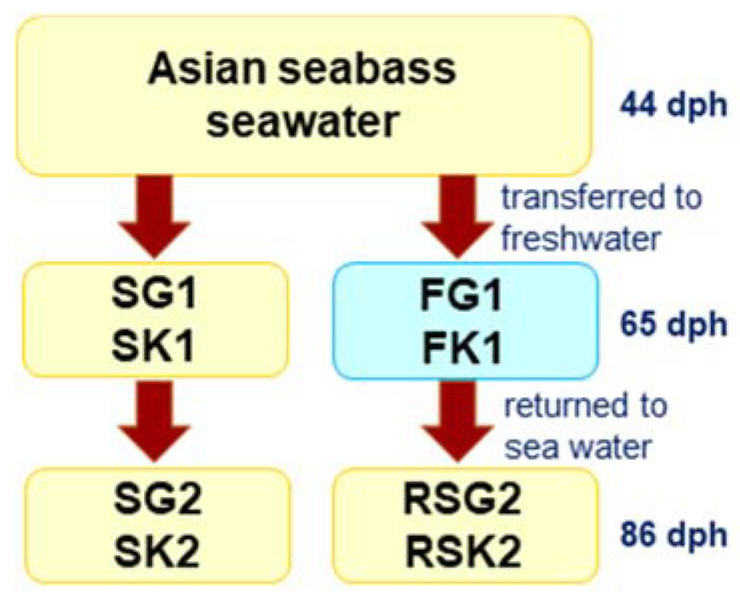
Flow chart outlining the experimental design for assessing the Asian seabass transcriptome under different salinity conditions. Abbreviations: dph: days post hatch; gills (SG1) and kidneys (SK1) from seawater grown fishes at 65 dph; gills (FG1) and kidneys (FK1) from freshwater grown fishes at 65 dph; gills (SG2) and kidneys (SK2) from seawater grown fishes at 86 dph; gills (RSG2) and kidneys (RSK2) from fishes returned to seawater at 86 dph.

Fish used for this experiment resulted from a regular mass-spawning event. Each such episode typically comprised of 5–10 males and females each and was conducted at the Marine Aquaculture Centre (MAC), St John’s Island, Singapore. Such mass-spawns commonly result in the creation of numerous full- and half-sib families, meaning that the cohort of fish analyzed was likely of mixed family status [45,46]. Briefly, fertilized eggs were incubated at a stocking density of 1000 eggs L^−1^ in 32 ppt seawater, where after upon hatching larvae were then placed into 10 L canvas tanks at a stocking density of 100 larvae L^−1^ (1000 larvae per canvas tank) where they were raised to 40 days post-hatch (dph). At 40 dph, ≈300 individuals of very similar size (≈< 0.2 g) were selected and transferred to the fish facility at the Temasek Life Sciences Laboratory, Singapore and were fed daily with TOMBOY Micro 80 (Skretting, Nutreco, Norway) feed. The fish were kept at 30–32 ppt for four days (44 dph) in order to acclimate, after which, the fish were randomly divided and transferred immediately into six separate tanks; three replicates for (i) freshwater (0 ppt) and three replicates for (ii) seawater (30–32 ppt) treatments. Each of the six tanks held ≈ 50 fish. At 65 dph (i.e., after 3 weeks of maintenance under the same salinity conditions), three individuals per tank were sacrificed and gill and kidney tissue samples collected from seawater-reared (gills: SG1; kidneys: SK1) and freshwater-reared (gills: FG1; kidneys: FK1) groups for histological and transcriptomic analyses. At this time point, a subset of fishes which had been reared in freshwater were transferred immediately into 32 ppt and grown for an additional 3 weeks and then sampled (gills: RSG2; kidneys: RSK2) (at 86 dph), whereas the rest from the seawater tanks were maintained there and sampled at the end (gills: SG2; kidneys: SK2; see Figure 1 for the experimental setup). Over the 42 days of the trial, no significant growth differences were found among seabass in the freshwater or saltwater treatments. In summary, for each sampling point, nine representative individuals each were collected (three from each replicate tank) for histology and RNA-seq analyses, respectively. Thus, in total, samples were collected from 72 individuals (RNAseq, 36; histology, 36). For sampling, the fishes were sacrificed by immersion in 2% tricaine, then dissected and kidney and gill samples collected. Samples for RNA-seq were snap-frozen in liquid nitrogen and stored at −80 °C prior to use. Samples collected for histology were stored in 10% buffered formaldehyde at 4 °C for at least 72 h.

Details of the experimental set-up are summarized as in Figure 1.

### 2.3. RNA Extraction, Library Construction, and Sequencing

Total RNA was extracted from organ samples collected using a RNeasy Mini kit with Dnase treatment (Cat No. 74106; Qiagen Singapore Pte. Ltd., Singapore), following the manufacturer’s protocol (three fish from each replicate tank). RNA concentration was determined using a NanoDrop 1000 (NanoDrop Technologies, USA). The RNA integrity number (RIN) of extracted total RNA was determined by running samples on an Agilent 2100 Bioanalyzer (Agilent Technologies, Nærum, Denmark). Total RNA was used to prepare libraries for sequencing using a TruSeq^®^ Stranded Total RNA Sample Preparation kit. The RNA-seq libraries were then sequenced with a read length of 75 nucleotides on the Illumina Nextseq 500 system. The average number of reads generated per sample was 46,169,877.

### 2.4. Mapping of Reads and Differential Expression Analyses

The amount of reads generated per sample along with percentage of reads were mapped to the Asian seabass reference genome [37] using Tophat v2.0.13, as summarized in Supplementary Table S1. Cuffdiff v2.2.0 was used for abundance calculation (represented as Fragments Per Kilobase of transcript per Million mapped reads (FPKM)) and identification of differentially expressed genes (DEGs). Criteria of at least two-fold difference, *p*-value <0.05 and false discovery rate (FDR) <0.1 was applied to identify the DEGs. The bioinformatics analyses reported here was performed as a paid service by DNA Link, Inc. (Seoul, South Korea).

### 2.5. Gene Ontology and Pathway Enrichment Analyses

GO annotation results of differentially expressed genes under different conditions were plotted using the Web Gene Ontology Annotation Plot (WEGO) (http://wego.genomics.org.cn). The gene names and the corresponding GO identities in the WEGO native format were used as input to the WEGO tool for GO classification and plots [47].

The web server, KEGG Orthology Based Annotation System (KOBAS 2.0) was used for identifying enriched pathways amongst the genes differentially expressed between kidneys and gills. This tool performed analyses in two steps: (1) annotation of the set of input genes with KO terms; (2) identification of statistical significance for each pathway. Fisher’s exact test and Benjamini–Hochberg FDR correction were used as parameters and a corrected *p*-value of <0.05 was used to identify the enriched pathways.

### 2.6. Histology

Kidney and gill samples fixed for 72 h in 10% chilled formalin were removed from the formalin and dehydrated in increasing gradients of ethanol (50%, 60%, 70%, 85%, 95%, and 100%). The dehydrated tissue was embedded in hydroxyethyl methacrylate (Historesin, Leica, Germany) and sectioned into a series of ≈8–10 sections per sample (section thickness: ≈5 µm), mounted on slides and colored using Hematoxylin-eosin (H/E). The gill sections were stained with Periodic Acid Schiff (PAS) [48], for the identification of mucus cells. The Von Kossa staining method [49] was used for the identification of chloride cells with calcium in the gill sections. Sections were stained with 1% silver nitrate for 20 min under the UV light, washed in water, immersed in 5% thiosulphate for 5 min to remove excess silver nitrate on the slide, washed in water, and stained with Hematoxylin for 5 min. Microscopic analyses were done using a Zeiss Axioplan 2 microscope mounted with a Nikon digital camera DXM 1200F (Oberkochen, Germany). In each case, nine representative fish per treatment were examined.

### 2.7. Counting Gill Chloride Cell Numbers and Kidney Glomerular Size

Chloride cell numbers were determined by counting the cells in a 0.075 mm^2^ gill surface area: five representative fishes were examined per treatment. Significant differences in the number of chloride cells and glomerular size between each group were determined by one-way ANOVA, followed by a Tukey HSD multiple pairwise comparison test using R™ version 3.4.1 [50]. Differences were considered significant only if their adjusted *p*-value (Padj) was <0.05 with 95% family-wise confidence level. The measurements of glomerular diameter were done using the Fiji software [51] (Supplementary Table S2).

## 3. Results and Discussion

The euryhaline nature of Asian seabass, a popular food fish in southeast Asia, Australia, and the Indian subcontinent, provides a useful model to study acclimation to varying environmental niches [37,38]. Under natural conditions, the Asian seabass are only between 12 dph to 12 weeks post-hatch (wph) when they have to first acclimatize to dramatic decreases in water salinity (marine to brackish/freshwater salinities) [40]; thus, young juveniles formed the basis of the study.

### 3.1. Changes in Gills and Kidney Histology Accompanied the Acclimation Response to Different Salinities

**Figure 2:**
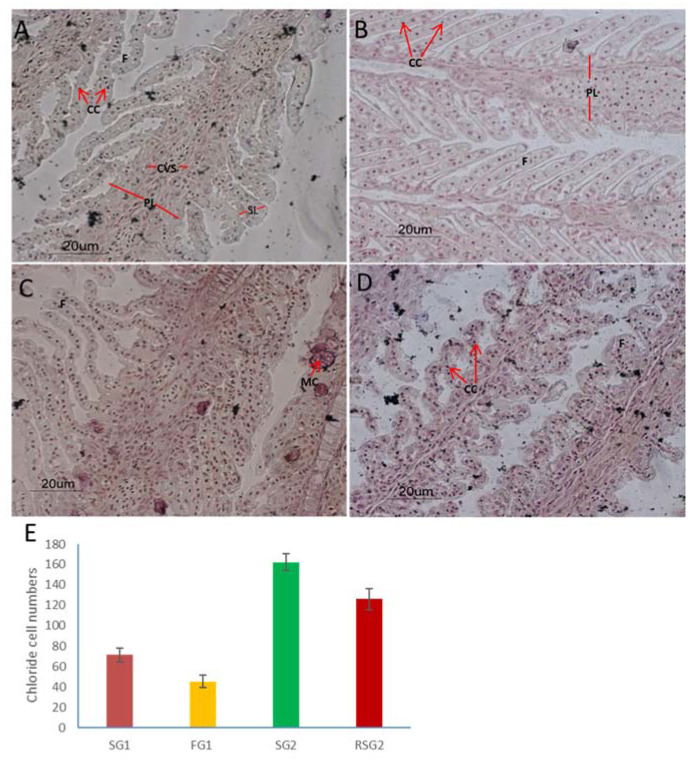
Histological analysis of Asian seabass gills showed a highly plastic phenotype with fishes transferred to freshwater showing a distinct decrease in chloride cell numbers which was regained upon being transferred back to seawater. Transverse section of the gills of Asian seabass (**A**) SG1, (**B**) FG1, (**C**) SG2, (**D**) RSG2. The sections were stained with Von Kossa staining. The scale bar is 20 µm. A multiple pairwise comparison test of chloride cell numbers between the four different groups showed that each group was significantly different (*p* < 0.05) from the other (**E**). Abbreviations: PL: primary lamella; SL: secondary lamella; CC: chloride cells; CVS: central venous sinus; MC: mucus cells; gills (SG1) from sea water grown fishes at 65 dph; gills (FG1) from fresh water grown fishes at 65 dph; gills (SG2) from sea water grown fishes at 86 dph; gills (RSG2) from fishes returned to seawater at 86 dph.

We performed a histological analysis of the gills and kidneys in order to ascertain the effect of changed salinity conditions in Asian seabass. Histology showed a large number of chloride cells on the gill lamellae and filaments in seabass kept in seawater in the two control groups (SG1 and SG2), as well as the seabass returned to seawater from freshwater (RSG2). In contrast, the numbers of chloride cells in seabass exposed to freshwater (FG1) was significantly reduced in comparison to those reared in seawater (Figure 2E; *p* < 0.05). This demonstrates that Asian seabass gill tissue undergoes substantive remodeling when exposed to a saline environment, resulting in increased proliferation of ionocytes as an acclimation requirement to excrete excess ions. When in freshwater these cells are required less and consequently their numbers reduce in the epithelial tissue.

This remodeling in Asian seabass is similar to that seen in two species of killifish, *Fundulus majalis* (a species which retains the ancestral salt tolerant phenotype) and *F. heteroclitus* (a species which exhibits osmotic plasticity) [20]. In this study, it was shown that the ability of the euryhaline killifish (*F. heteroclitus*) to acclimate to salinity changes is largely attributed to its dynamic capacity to remodel the morphology of the gill epithelium. This involved, similar to our observations, a drastic reduction in the chloride cell numbers in the gills of freshwater grown fishes which were regained upon transfer of fish back into seawater [20].

**Figure 3:**
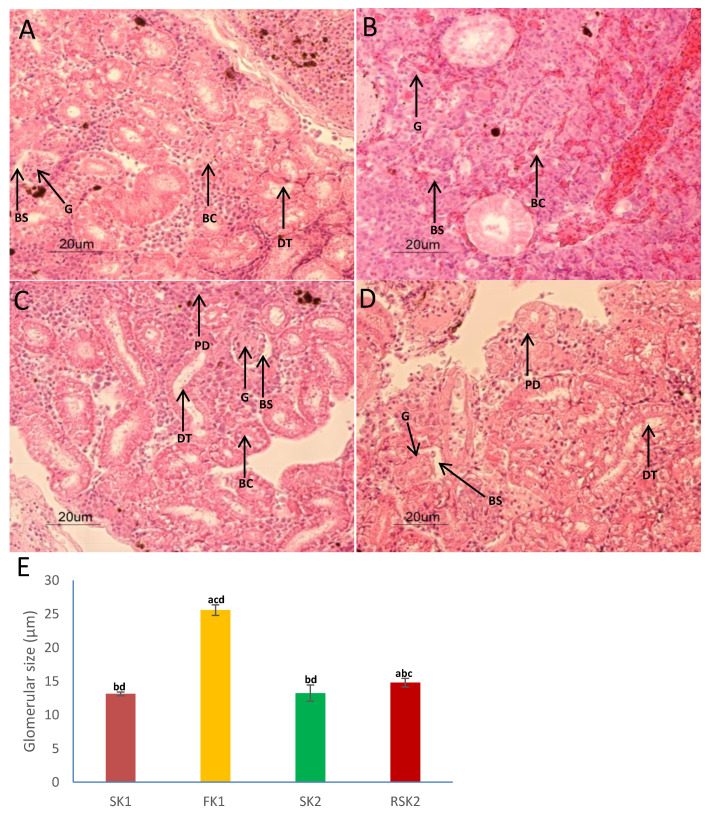
Histological analysis of Asian seabass grown in seawater shows structural changes in the Bowman’s capsule, glomerulus, and papillary ducts of the kidney upon being transferred and grown in freshwater. Transverse section of the Asian seabass kidney (**A**) SK1, (**B**) FK1, (**C**) SK2, (**D**) RSK2. The sections were stained with H&E. The scale bar is 20 µm. Multiple pairwise comparison test of glomerular size between the four different groups (**E**). The letters above each bar (a: SK1; b: FK1; c: SK2; d: RSK2) indicates the groups from which it is significantly different (*p* < 0.05). Abbreviations: BS: Bowman’s space; G: glomerulus; DT: distal tubules; kidneys (SK1) from seawater grown fishes at 65 dph; kidneys (FK1) from freshwater grown fishes at 65 dph; kidneys (SK2) from seawater grown fishes at 86 dph; kidneys (RSK2) from fishes returned to seawater at 86 dph.

Histological analyses of Asian seabass kidney revealed collecting tubules of varying shapes and sizes located throughout the kidneys as well as glomeruli surrounded by Bowman’s space with interspersed hematopoietic regions (Figure 3). Kidneys of seabass individuals adapted to freshwater had well developed glomeruli (≈25 µm), whereas, glomerulus size was visibly reduced in the kidneys of the seawater controls (≈13 µm; SG1 and SG2), as well as the group which had been returned to seawater after being grown for 3 weeks in freshwater (RSG2). Due to the shrunken glomeruli in these fishes, the Bowman’s space was also more evident. These features were more distinct in fish kept in seawater for longer duration (SG2 vs. SG1) (Figure 3). In addition, structures within the kidneys such as the collecting tubule and glomeruli were compact and well developed in seabass acclimated to freshwater. Fish grown in a hypotonic environment need to pass large volumes of urine in order to maintain homeostasis, hence typically have well developed glomeruli [52,53]. Further, it has been reported that decreasing the size and number of glomeruli (i.e., *Trisopterus luscus*), or secondary elimination of glomeruli in certain fish species (i.e., *Syngathidae, Batrachoididae*) serves as a protective mechanism to limit fluid loss in the marine environment [9]. This reduction/elimination of glomerular surface area possibly decreases fluid motion which in turn allows more time for passive diffusion of water back into the blood [5,9,52,53].

### 3.2. RNA-Seq Analyses

**Figure 4:**
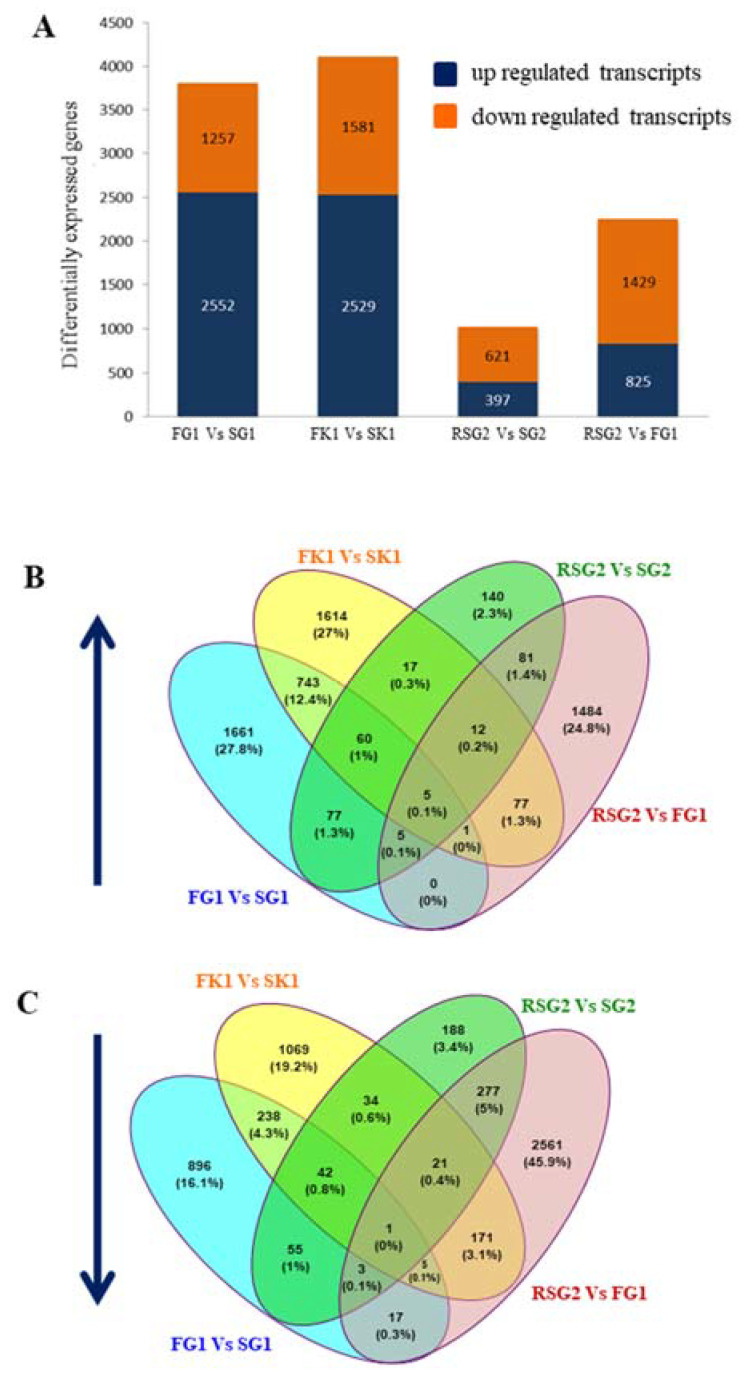
Summary of differentially expressed genes in the gills and kidneys of Asian seabass acclimated to different salinity conditions. (**A**) The graph shows the number of differentially expressed genes (up- and downregulated) under different conditions—FG1 vs. SG1, FK1 vs. SK1, RSG2 vs. SG2, and RSG2 vs. FG1. (**B**,**C**) Venn diagrams showing the unique differentially expressed genes as well as genes shared between the different salinity conditions or organs. Abbreviations: Refer to Figure 1.

RNA-Seq of gill and kidney tissues were performed to investigate gene regulation in these two osmoregulatory tissues under different salinities. A large number of differentially expressed (DE) transcripts were observed in the two major osmoregulatory organs: kidneys (4110) and gills (3809) of fishes adapted to freshwater compared to seawater-grown fishes (FG1 vs. SG1; FK1 vs. SK1). For both gills and kidneys, freshwater fish in comparison to saltwater samples exhibited a larger proportion of upregulated transcripts (2552: gills; 2529: kidneys), compared to downregulated transcripts (1257: gills; 1581: kidneys). Conversely, gills of seabass reared in seawater (SG2) when compared to gills of fish grown in freshwater (RSG2) before transfer to seawater for 3 weeks showed the least number of differentially expressed transcripts (RSG2 vs. SG2 (1018); up: 397, down: 621). An intermediate number of differentially expressed transcripts could be identified on comparing the gills of seabass adapted to freshwater at 65 dph and gills of fishes which had been returned to seawater and grown for an additional 3 weeks (RSG2 vs. FG1 (2254); up: 825, down: 1429) (Figure 4).

In addition, we identified transcripts which were unique to, or showed overlap between, different salinity conditions. Amongst the upregulated transcripts identified in the four pairwise comparisons, a larger percentage of transcripts were unique to a specific set of comparisons (FG1 vs. SG1: 27.8%, FK1 vs. SK1: 27%, and RSG2 vs. FG1: 24.8%). This trend was also observed within the downregulated dataset, with the majority differentially expressed transcripts unique to RSG2 vs. FG1: 45.9%, FK1 vs. SK1: 19.2%, and FG1 vs. SG1: 16.1%. The least number of unique differentially expressed transcripts were seen in RSG2 vs. SG2 (unique upregulated: 2.3%; downregulated: 3.4%; Figure 4). Additionally, the largest common set of differentially expressed transcripts was shared between the gills and kidneys—FG1 vs. SG1 and FK1 vs. SK1; Totally—1095; 809 upregulated and 286 downregulated (Supplementary Table S3).

### 3.3. The Euryhaline Trait is Pliable

**Figure 5:**
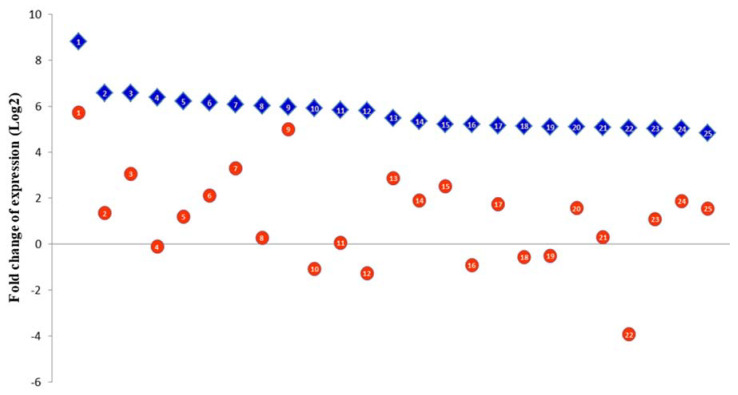
The euryhaline trait is pliable and involves the differential expression of a core set of genes. The top 25 differentially expressed genes in the FG1 vs. SG1 comparison (blue diamonds; 1–25) were checked for in RSG2 comparison vs. SG2 (orange circles; 1–25). The majority of these transcripts were still upregulated in RSG2 compared to SG2 and all of them were expressed to a much lesser extent compared to the FG1 vs. SG1. Thus, the top 25 upregulated genes (FG1 vs. SG1) seem to be reinstating themselves to the saline conditions in the RSG2 group of fishes. Abbreviations: Refer to Figure 1.

In order to assess the flexibility of the euryhaline trait, the transcriptome of seabass which had been returned to seawater after having adapted to freshwater was compared to the transcriptome of seabass which had been raised in seawater throughout the trial (both groups were of 86 dph age at the time of sampling). This pairwise comparison (i.e., SG2 vs. RSG2) had the least number of differentially expressed genes (both up- and downregulated), indicating that the fishes quickly reverted to the seawater-adapted gene regulatory state upon being transferred back to seawater. A closer look at the upregulated genes further made it apparent that the gene expression profile of the seabass in the RSG2 group was reinstating itself to the physiological state required by fishes for survival under saline conditions. This was evident upon comparing the top 25 differentially expressed transcripts in the FG1 vs. SG1 to the differentially expressed transcripts identified in the RSG2 vs. SG2 comparison. All these transcripts were expressed at a higher level in the FG1 vs. SG1 comparison compared to saline re-acclimated vs. continually seawater-maintained seabass. Additionally, eight of the top 25 transcripts could be identified on the list of top 100 upregulated transcripts in the RSG2 vs. SG2 comparison. This included thyroglobulin (Tg) which was the most upregulated transcript across both the comparisons; however, it was ≈ 445-fold upregulated in FG1 vs. SG1 and only 50-fold upregulated in RSG2 vs. SG2 (Figure 5). Thus, when the fishes were returned to seawater, the transcriptome of fishes was pliable enough to transform quickly back from the freshwater to seawater state.

## 4. GO and Pathway Analyses

**Figure 6:**
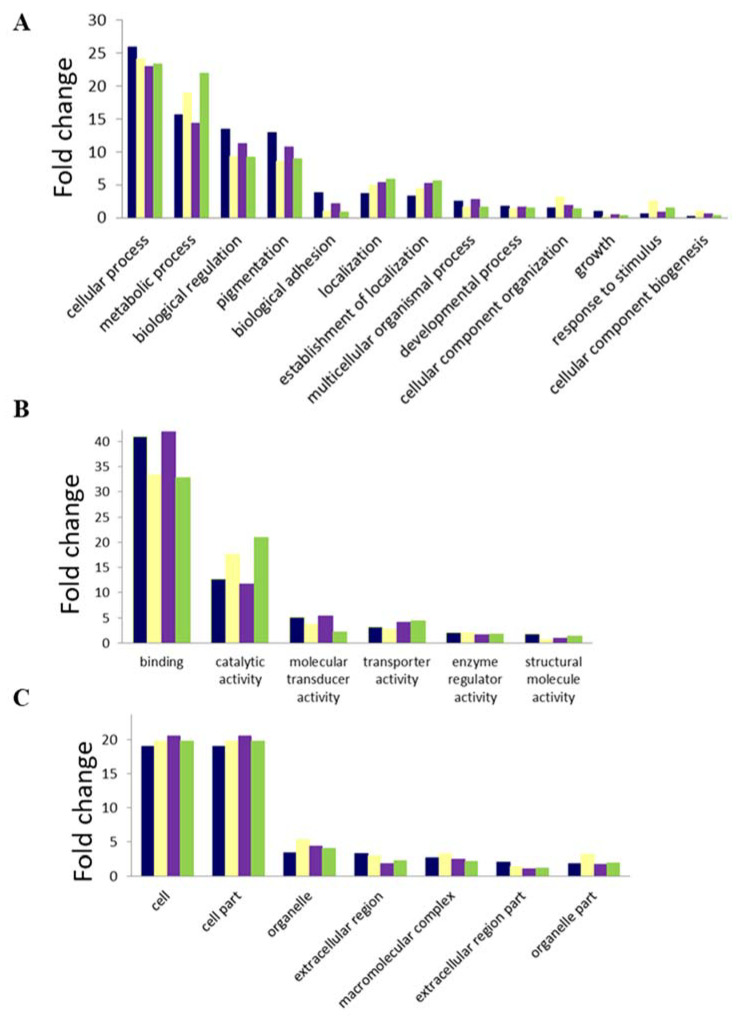
Gene ontology analyses of (**A**) Biological processes, (**B**) molecular Functions, and (**C**) Cellular components using the Web Gene Ontology Annotation Plot (WEGO) of the Asian seabass gill and kidney transcriptomes under different salinity conditions. Only categories with 15 or more protein associations are shown. Blue: upregulated in FG1 vs. SG1; Yellow: downregulated in FG1 vs. SG1; Purple: upregulated in FK1 vs. SK1; Green: downregulated in FK1 vs. SK1. Abbreviations: Refer to Figure 1.

Differentially expressed genes were assigned Gene Ontology (GO) terms under the following categories: Biological process, Molecular function, and Cellular component. The following comparisons were made: FG1 vs. SG1-upregulated, FG1 vs. SG1-downregulated, FK1 vs. SK1-upregulated and FK1 vs. SK1-downregulated. Notably, for all the differential gene expression comparisons, the same set of GO terms was enriched both for upregulated and downregulated transcripts and for each of the three categories, albeit to a different extent in each case. The most enriched biological processes for the differentially expressed genes in both the kidney and gill transcriptomes under changed salinity conditions were cellular process, metabolic process, biological regulation, and pigmentation. Amongst the molecular functions, the most enriched were ‘Binding and catalytic activity’, while ‘Cell and cell part’ were most represented amongst the cellular component ontologies (Figure 6). All the GO terms identified for the three categories in our study were also identified as important (with the exception of ‘Growth’) by a previous NGS-based transcriptomic comparison of freshwater- and brackish water-grown *P. altivelis* larvae [24].

A large number of pathways (15) were exclusively enriched in the seabass gills under varied salinity conditions. These included pathways related to ‘Amino acid metabolism’, ‘Organismal systems’ (endocrine, digestive and excretory system), ‘cAMP signaling pathway’, and ‘Human disease’ (hypertrophic cardiomyopathy (HCM), dilated cardiomyopathy, amoebiasis, and pathways in cancer). Only two pathways overlapped between the gills and kidneys: ‘ECM receptor interaction’ and ‘Protein digestion and absorption’. In addition to ‘Human disease’ and ‘Organismal systems’, several pathways representing environmental information processing such as ‘cGMP-PKG signaling pathway’, ‘P13-Akt signaling pathway’, and ‘Cell Adhesion Molecules (CAM)’, as well as ‘Cellular Processes’ (focal adhesion) were exclusive to the kidneys. Transcriptome profiling of *Oreochromis niloticus* hepatopancreas at different salinities, for example, identified 1852 differentially expressed genes related to ‘Metabolism’ (lipid, energy, protein), ‘Immune system’, ‘Cell-activity’, and ‘Signaling pathways’ [28].

### 4.1. Genes Involved in Osmoregulation, Tissue/organ Morphogenesis and Regulation of Cell Volume Are Important for Salinity Acclimation

Ion transporters are central to osmoregulation in fishes [22,23]. Profiling the euryhaline transcriptome of *Mozambique tilapia* identified a large number of transcripts (>100) related to inorganic ion channels and transporters expressed both in fresh and saltwater exposed gills [8]. Additionally, a study on the euryhaline killifish reported drastic changes in the osmoregulatory organs at the morphological level, as well as differential expression of genes involved in osmotic stress signaling, ion transport, and regulation of cell volume [20]. Similarly, in our study, several genes important for ion re-adsorption and electrolyte homeostasis, including sodium/hydrogen exchangers and ion transporters (e.g., Na^+/^K^+^-ATPases) were represented in the set of differentially expressed genes in the gills and kidneys.

**Figure 7:**
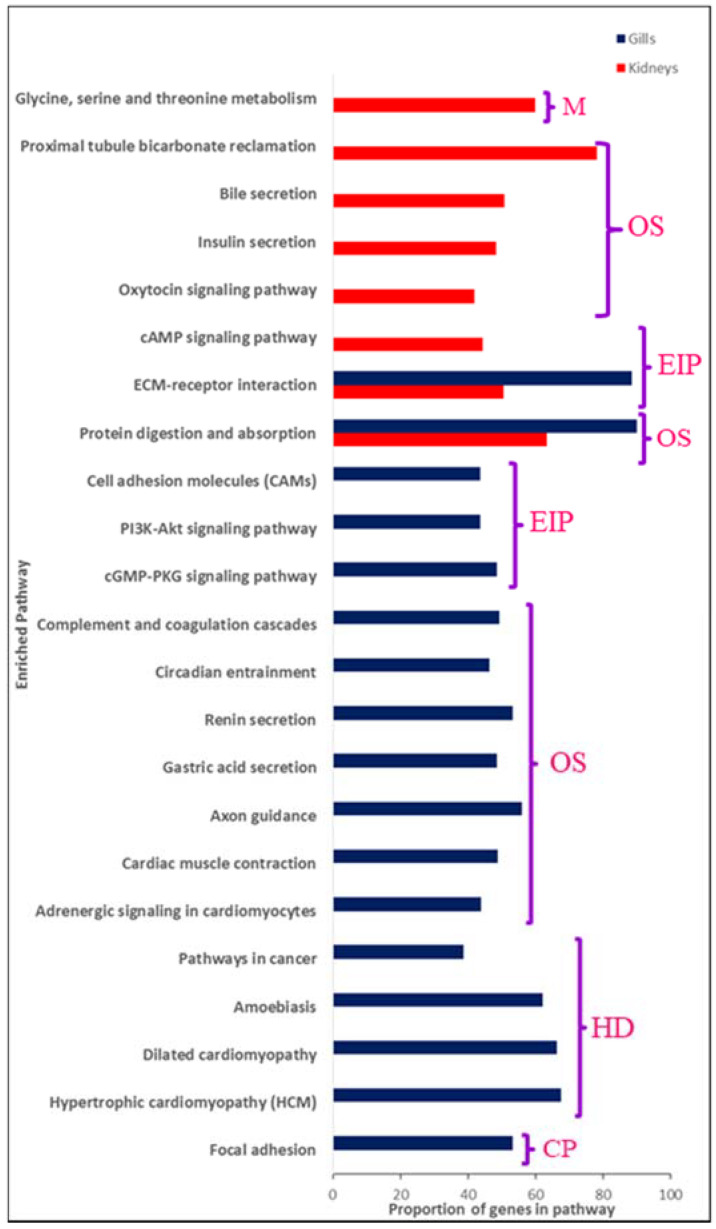
Pathways significantly (*p* < 0.05) enriched amongst the differentially expressed transcripts in the osmoregulatory organs. The pathway is indicated on the *Y*-axis and the proportion of genes in the pathway is indicated on the *X*-axis. Statistical significance was determined as Benjamini–Hochberg adjusted *p*-value < 0.05. Abbreviations: M: Metabolism; OS: Organismal system; EIP: Environmental information processing; HD: Human disease; CP: Cellular processes. Additional information can be found in Supplementary Table S5.

In addition, several genes coding for the collagen family of proteins were amongst the most differentially expressed transcripts in the gills, as well as kidneys (Supplementary Table S4). These together with several other genes represented the ‘ECM (extracellular matrix)–receptor interaction’ pathway; one of the significantly enriched pathways both in the gills as well as the kidneys transcriptomes (Figure 7). The extracellular matrix plays a pivotal role in tissue and organ morphogenesis and the differential expression of the genes belonging to this pathway would probably be needed for the rapid remodeling of the gills and kidneys as part of the osmo-adaptive response.

An interesting pathway significantly enriched exclusively in seabass kidneys was linked to the excretory system: ‘Proximal tubule bicarbonate reclamation’. A dominant function of the kidney proximal tubule is to secrete acid in the tube lumen, this helps in reabsorbing the majority of bicarbonate ions (HCO3(-)) and regulating the blood pH. This pathway was represented by several sodium/potassium-transporting ATPases (ATP1A1, ATP1A3, ATP1B1, ATP1B2, and ATP1B3). Na+/K+-ATPase are membrane proteins which function as pumps, playing a key role in maintaining a Na^+^ and K^+^ ion electrochemical gradient across the plasma membrane, necessary for osmoregulation. The enzyme is composed of a large catalytic Alpha subunit (encoded by numerous genes) and a smaller glycoprotein beta subunit [54,55,56]. In addition, solute carrier proteins such as SLC9A3 known to have a role in balancing pH [57] were also found to be upregulated in the kidneys of fish growing in seawater conditions and were also represented in this pathway. The proximal tubule bicarbonate reclamation genes were more highly expressed in the kidneys of saltwater grown fish compared to freshwater reared, reflecting the necessity to excrete excessive ions and osmoregulate under saline conditions.

A pathway linked to amino acid metabolism, ‘Glycine, serine, and threonine metabolism’, was also uniquely enriched in seabass kidneys. Amino acids have been previously shown to be of significance for salinity acclimation, either as an energy source, or as osmolytes for regulating cell volume [58]. Alterations in amino acid levels in response to salinity have been described earlier for several animals such as mussels [59], crabs [60,61], insect larvae [62], and fish [63,64]. Increased salinity coincides with an increase in amino acid levels and has been observed also in the euryhaline Senegalese sole and other teleost fishes [65]. In our study too, the genes coding for several enzymes in the amino acid metabolism pathway were upregulated in the kidneys of fishes reared in seawater vis-à-vis freshwater.

### 4.2. Ion Loss under Freshwater Conditions Seems to Be Restricted by Upregulation of Several Tight Junction Proteins

A number of organismal system pathways were also observed to be enriched, of which one pathway was linked to environmental acclimation; ‘Focal adhesions’. Focal adhesions are cell-matrix adhesions wherein actin bundles are secured to transmembrane receptors from the integrin family [66,67,68]. This pathway was represented by several genes coding for collagen and myosin families of proteins and were amongst the top upregulated transcripts in the gills of freshwater grown seabass compared to seawater. Another pathway specifically enriched in the gills was the ‘Cell adhesion molecule’ (CAM) pathway. CAMs are glycoproteins present on the cell’s surface and are implicated in a wide array of biological functions such as cell proliferation, differentiation, motility, trafficking, apoptosis, and tissue architecture [69,70]. A large number of diverse cell adhesion molecules were represented in the CAM pathway which included several members from the integrin, cadherin, and claudin family of proteins (Supplementary Table S6). An important physio-regulatory mechanism operational in freshwater is to limit ion loss instead of actively taking up the ions from the surrounding environment. Claudins are tight junction proteins which can form these ion-selective pathways. Differential expression of claudins has been shown to be important for permeability changes associated with acclimation to the freshwater environment. For example, hypo-osmotic acclimation in *Fundulus* (killifish) involves the upregulation of at least two claudins, CLDN4 and CLDN7, along with tight junction proteins, e.g., desmocollin 1 (DSC1), connexin-32.2 (cx32.2), occludin (OCLN), and periplakin (PP) [20]. The absence of a reference genome for *Fundulus* along with incomplete representation of the full claudin family on the microarray used for the study indicate that there could be more claudins involved in the osmotic acclimation of this euryhaline species [20]. In our study, 14 claudins were identified as differentially expressed in comparisons of fresh and saltwater gill transcriptomes. Of these, 11 (including *cldn7*) were upregulated in gills of seabass adapted to freshwater conditions. The majority of the claudins were represented in the CAM pathway.

### 4.3. Genes Rrequired for Growth and Development Were Upregulated under Freshwater Conditions

Salinity, a critical environmental factor, is known to affect growth rate in teleosts, chiefly by altering metabolic rate and hormonal stimulation [71,72,73,74]. Indeed, a large number of migratory fishes experience drastic changes in body functions, such as smoltification in salmonids, or silvering in eels, preceding the migration into a dramatically different osmotic environment. Thus, the ontogenic change in euryhaline fishes during early life stages and during maturation is an important aspect to consider in studying euryhalinity [7]. Genes coding for proteins required for bone (osteocalcin) and teeth (fibulin-7) development were amongst the topmost DEGs upon exposure of Asian seabass juveniles to freshwater [75]. Possibly, the freshwater serves as a cue for growth and development since in the wild, older juveniles commonly migrate to the freshwater where they grow and mature. Indeed, it has been observed that Asian seabass grown in freshwater do well and are less prone to disease outbreaks compared to their seawater-grown counterparts (Susan Queh, personal communication).

### 4.4. Genes Coding for Mucins Were Upregulated under Saline Conditions

The gills also have mucous cells which are large modified, mucous-secreting, columnar epithelium cells [76]. Several genes coding for mucins were found to be upregulated in gills of fishes growing in seawater compared to freshwater. Previous studies have similarly shown an increase in mucous production in response to salinity [77], disease [78], as well as pollutants [79,80], air exposure, and desiccation stress [81,82,83,84,85,86,87]. Increase in mucous cell numbers and mucous secretion has been reported to be a protective mechanism against changes in ionic homeostasis and by possibly reducing water loss at the gill surface [78,88,89]. The mucous also serves as an important protective mechanism against possible injuries, disease causing bacteria, viruses, and pollutants [77,78,90,91].

### 4.5. Hormones Play an Important Role in Regulating Euryhalinity

Hormones play an important role in maintaining the osmotic and ionic balance in euryhaline fishes. Several hormones have been implicated in euryhaline acclimation such as adrenocorticotrophic hormone (ACTH), prolactin, growth hormone, insulin-like growth factor-1 (IGF-1), cortisol, thyroxine, triiodothyronine, and angiotensins [7,92]. Prolactin and growth hormone/insulin-like growth factor-1 can be categorized as slow acting hormones which help to acclimatize the body for a long-term response by altering the numbers of ion transporters, ionocytes and other osmoregulatory cells [7]. On the other hand, angiotensin (AGT) is a fast-acting hormone which helps by regulating the drinking rate and the activity of ion transporters in the fish osmoregulatory organs [7]. AGT was represented in one of the significantly enriched pathways; ‘Renin secretion’. Renin is central to regulation of extracellular fluid volume and maintenance of blood pressure. Typically in response to lowering of blood pressure or decreased sodium levels, renin secretion is stimulated, upon which it cleaves angiotensinogen (AGT) to yield angiotensin I, this is further converted to angiotensin II by angiotensin I converting enzyme (ACE). Prolactin receptor (PRLR), growth hormone receptor (GHR), and insulin-like growth factor-1 (IGF1) were represented in the ‘PI3K-Akt signaling’ pathway, an environmental information processing pathway regulating core cellular processes such as transcription/translation, proliferation, growth, and survival. Both the Renin secretion and PI3K-Akt signaling pathways were enriched among the upregulated transcripts in the gills of Asian seabass reared in freshwater compared to those grown in saltwater (FG1 vs. SG1 comparison).

### 4.6. Circadian Entrainment Could Be Important for Establishing Euryhalinity

Temporal adaptations in animals rely on an internal physiological clock that is constantly reset with light and other stimuli to synchronize them with their environment. Secretion of melatonin in the night by the pineal gland, a neuroendocrine organ in vertebrates, is a major output from the clock gene network [93]. The fish pineal organ is mainly regulated by light, however, environmental variables such as temperature and salinity have been shown to affect melatonin levels in fishes [94,95,96]. In turn, melatonin has been suggested to have a role in osmoregulation, appetite regulation, and smolting time in fishes [95,97,98,99]. Besides this, there is a pathway called ‘Circadian entrainment’ which is represented by genes involved in routing the internal biological clock in response to environmental cues [100,101]. Gills of Asian seabass adapted to freshwater showed an enrichment of genes belonging to this pathway. Apart from light, melatonin plays an important role in entrainment by inhibiting the effect of light via adenylate cyclase inhibition. The various entrainment pathways act through regulating CAMP responsive element binding protein (CREB), the phosphorylated CREB in turn activates the expression of clock genes [102,103,104]. This included three members of the Period family of genes (PER1, PER2, and PER3) and Melatonin Receptor 1A (MTNR1A), one of the two receptors for melatonin. The genes belonging to the PER family, as well as the melatonin receptor family, are important for maintaining circadian rhythms associated with movement, metabolism and behavior. In freshwater reared Asian seabass, CREB1, PER1, PER2, PER3, MTNR1A, and all the other genes representing the circadian entrainment pathway were specifically upregulated in gills.

The effect of salinity on melatonin levels have been previously described for fishes such as gilthead sea bream (*Spaurus aurata*), European seabass (*Dicentrarchus labrax*), and rainbow trout (*Oncorhynchus mykiss*) [95,97,105]. In the first two species, melatonin levels decreased in seawater compared to freshwater (similar to what we observed for Asian seabass in our study), whereas the converse was seen in rainbow trout; melatonin levels increased under saline conditions. The association of melatonin levels with salinity changes seems to have been observed exclusively for euryhaline fish species. This indicates an important role for circadian entrainment not only for mediating the salinity response, but also for fish migration which is an inherent feature of these fishes. This could additionally explain why the effect of salinity varied depending upon the fish species in question, with rainbow trout being an anadromous fish, whereas seabream, Asian seabass, and European seabass are catadromous fishes.

## 5. Conclusions

Euryhalinity is manifested as a plastic trait in Asian seabass, orchestrated by complex physiological processes which are apparent at all levels of biological organization from behavior to molecules. Thus, when salinity is low, juvenile Asian seabass physio-regulate more like freshwater fishes, and when salinity is high, they physio-regulate like marine fishes. This was evidenced in Asian seabass in the various salinity treatments reported in the present study, both at the transcriptome level, as well as through the extensive cellular remodeling visualized at the histological level. A large number of transcripts representing osmo-adaptive pathways were differentially expressed. The interesting additional aspect of the study, however, was the finding of an association of growth as well as circadian entrainment with fishes grown in freshwater. Euryhaline fishes such as the Asian seabass are typically migratory. Therefore, this finding presents the possibility that this could well be a feature of these fishes, with freshwater serving as a cue to route the biological clock both for growth, as well as for migration necessary for accomplishing this phase of the species’ lifecycle. However, these findings are only the first step in the identification of putative genes of interest which could play an important role in mediating euryhalinity response in Asian seabass and further studies are needed to ascertain the exact role of these genes and pathways in salinity acclimation. In addition, it may be useful to examine consistency of this acclimation response in other genetic populations of seabass to detect if the response is uniform despite the genetic background. Furthermore, it is important to note that there are a large number of different tools for both mapping as well as differential gene expression analyses. Use of these tools in different pipelines are expected to give somewhat different results. For instance, some pipelines may be permissive and give a high rate of false positives whereas others might be more stringent and may miss out on potentially useful genes. In our study, Cuffdiff2 was used for identifying the DEGs. A study on the comparison of edgeR, DESeq, and Cuffdiff2 showed a high degree of overlap between the DEGs identified by the three tools [106]. It was also observed that edgeR performed better than the other two tools in terms of its ability to identify true positives. However, upon data simulation, the higher number of identified DEGs coincided with the most number of false positives detected using edgeR (108) in comparison to Cuffdiff2 (77) and DeSeq (8) [106]. Nevertheless, few studies have shown that cuffdiff is generally more conservative in comparison to other bioinformatics pipelines used for identifying DEGs [106,107]. It is, therefore, possible that our analysis may have failed to capture some of the changes in gene expression. However, notwithstanding the expected variations in the usage of different tools, the data generated in this study does provide useful inventory of the Asian seabass transcriptome in response to salinity changes.

## Data Availability

The sequences have been submitted to DDBJ/EMBL/NCBI GenBank under the accession SRP248604.

## Supplementary Materials

The following are available online at https://www.mdpi.com/2073-4425/11/7/733/s1, Table S1: Summary statistics of the RNA-Seq data and mapping rates to the annotated Asian seabass genome, Table S2: Chloride cell numbers present in gills and kidney glomerular size, Table S3: List of transcripts common to FG1 Vs SG1 and FK1 Vs SK1 comparisons (Up as well as down regulated), Table S4: List of genes coding for collagen family of proteins identified amongst the most differentially expressed transcripts in the gills and kidneys, Table S5: Pathways enriched in the Asian seabass gills and kidneys, Table S6: List of cell adhesion molecules represented in the CAM pathway specifically enriched in the gills.
